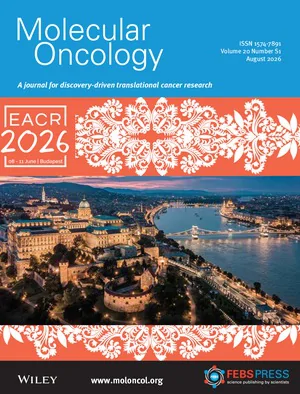# Abstracts

**DOI:** 10.1002/1878-0261.70318

**Published:** 2026-08-23

## Abstract

Abstracts submitted to the ‘EACR 2026 Congress: Innovative Cancer Science’, from 08–11 June 2026 and accepted by the Congress Organising Committee are published in this Supplement of *Molecular Oncology*, an affiliated journal of the European Association for Cancer Research (EACR).